# Isometric muscle training of the spine musculature in patients with spinal bony metastases under radiation therapy

**DOI:** 10.1186/1471-2407-11-482

**Published:** 2011-11-09

**Authors:** Harald Rief, Alexandra D Jensen, Thomas Bruckner, Klaus Herfarth, Jürgen Debus

**Affiliations:** 1 Dept. of Radiation Oncology, INF, University of Heidelberg Medical School, Heidelberg, Germany

## Abstract

**Background**

Osseous metastatic involvement of the spinal column affects many patients with a primary tumour disease of all entities. The consequences are pain both at rest and under exertion, impairments in going about day-to-day activities, diminished performance, the risk of pathological fractures, and neurological deficits. Palliative percutaneous radiotherapy is one of the therapeutical options available in this connection. The aim of this explorative study is to investigate the feasibility of muscle-training exercises and to evaluate the progression- and fracture-free survival time and the improvement of bone density, as well as to assess other clinical parameters such as pain, quality of life, and fatigue as secondary endpoints.

**Methods/Design**

This study is a prospective, randomized, monocentre, controlled explorative intervention study in the parallel-group design to determine the multidimensional effects of a course of exercises at first under physiotherapeutic instruction and subsequently performed by the patients independently for strengthening the paravertebral muscles of patients with metastases of the vertebral column parallel to their percutaneous radiotherapy. On the days of radiation treatment the patients in the control group shall be given physical treatment in the form of respiratory therapy and the so-called "hot roll". The patients will be randomized into one of the two groups: differentiated muscle training or physiotherapy with thirty patients in each group.

**Discussion**

The aim of the study is to evaluate the feasibility of the training programme described here. Progression-free and fracture-free survival, improved response to radiotherapy by means of bone density, and clinical parameters such as pain, quality of life, and fatigue constitute secondary study objectives.

**Trial Registration**

ClinicalTrials.gov: NCT01409720

## Background

Osseous metastatic involvement of the spinal column affects many patients with a primary tumour disease of all entities. In many cases this involvement is indicative of a progressed stage of a primary malignant disease. 70 percent of all patients who die of the sequelae of the tumour disease exhibit bone metastases [1]. 80 percent of all osseous metastases originate from mammary, prostatic, bronchial, renal-cell, or thyroid carcinomas [2]. In men, the primary tumour in 60 percent of all cases is a carcinoma of the prostate [3], among women, it is in 70 percent of the cases a mammary carcinoma. 30 percent of all metastases of the skeletal system and ten percent of all primary bone tumours are found in the spinal column. The former are located in the lumbar (52%), thoracic (36%) and cervical column (12%) [4]. The consequences are pain both at rest and under exertion, impairments in going about day-to-day activities, diminished performance, the risk of pathological fractures, and neurological deficits. Pain is the essential factor for the decrease in the quality of life of patients with bone metastases [5]. Pathological fractures occur in 5% and compressions of the spinal cord in 10-15% of all patients [6]. In its role as the central axial organ, the spinal column stands in the focus of all mobility options of the individual patient and, when compromised, constitutes a mobility-restricting factor. The generation of power by the paravertebral muscles and the patient's mobility correspondingly play a decisive role regarding his/her quality of life. In their study involving patients with an advanced tumour disorder, Cheville et al. [7] were able to demonstrate that physiotherapy can be performed parallel to radiotherapy with a beneficial effect. The primary intention of this study is to review the feasibility of the project. Strengthening the paravertebral muscular system is reported to have a positive effect not only on the sensation of pain, but also on the quality of life and fatigue. Isometric training of the paravertebral muscles is anticipated to produce a raised perfusion of the segments of the vertebral column involved. This aspect may, in combination with percutaneous radiotherapy, result in a better response to therapy. The potentially raised risk of fracture in combination with an injury of the spinal cord involving neurological deficits results in patients suffering a constant state of anxiety of varying distinction. In most cases, this fear of such a serious event in turn results in an unintended "vicious circle" comprising immobility, pain, and ever-decreasing physical performance. Pain, anxiety, and impaired physical mobility are in virtually all cases associated with a reduced quality of life and frequently result in negative consequences for the patients' participation in society. This controlled combination therapy may relieve the negative effects of the tumour disease for the patients. The literature has so far not described any dedicated ergotherapeutical measures employing isometric muscle training in connection with bone metastases. The extent to which specific, regular, and differentiated training of the paravertebral muscle system can be performed may be jeopardized by the reduced general condition of the patients, their pain situation, and their fear of suffering fractures, which is why the feasibility of the study poses the greatest challenge. In terms of the localization and pain symptoms, these are similar to the complaints experienced in osteoporosis and vertebral-disk syndromes. Among these patients there are numerous indications of the positive effect of targeted physical exercise on pain and mobility [8-11]. The findings of this direction of research shall also be considered in this study. Patients undergoing treatment in the form of percutaneous radiotherapy who from the orthopedic and radiological aspects are classified as not being at risk of suffering fractures will receive a second component of therapy involving isometric training of the paravertebral muscle system.

## Methods/design

**Figure 1:**
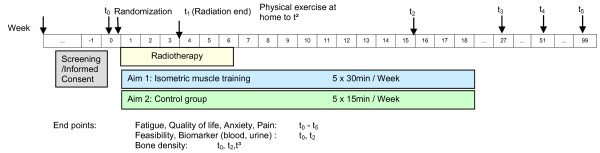
**Flowchart of the study**.

This study is a prospective, randomized, monocentre, controlled explorative intervention study in the parallel-group design to determine the multidimensional effects of a course of exercises at first under physiotherapeutic instruction and subsequently performed by the patients independently for strengthening the paravertebral muscles of patients with metastases of the vertebral column parallel to their percutaneous radiotherapy (Figure 1). Since the osseous metastases of the individual patients may be located at different levels of the vertebral column, three different types of exercises have been selected to ensure an even isometric training of all vertebral-column muscles. On the days of radiation treatment the patients in the control group shall be given physical treatment in the form of respiratory therapy and the so-called "hot roll". The plan foresees the recruitment, over a period of twelve months, of a total of 60 patients with metastases of the vertebral column who are scheduled to undergo radiotherapy. Prior to their enrolment into the study, the patients will undergo a staging of the vertebral column in connection with their radiation-planning CT to measure the bone density. After the baseline results have been recorded, the patients will be randomized into one of the two groups: differentiated muscle training (n = 30) or physiotherapy (n = 30). The interventions will start at the same time as radiotherapy, taking place on each day of irradiation (Mondays thru Fridays) for a period of two weeks. Each sport intervention will last approx. 30 minutes, the physiotherapy measure approx. 15 minutes over a two-week period. After the end of the radiotherapy/after two weeks, the patients participating in the training group will continue to do the exercises started under the instruction of the therapist on their own at their homes, recording the exercises and the pain load in a daily protocol report. The participants in the control group will not do any exercises or measures at home. The target parameters will be measured and recorded at the end of the irradiation period (t_1_) and twelve weeks (t_2_) and six months following the end of the irradiation period (t_3_). Follow-up measurements are scheduled to take place twelve, 18, and 24 months after the end of irradiation.

## Recruitment and randomization

The patients will be given information on the study by the medical personnel of the Radiotherapy Department in connection with the planning of the radiotherapy regimen (approx. 1-2 weeks prior to the start of radiotherapy). If they are interested in participating in the study, the potential study candidates will be given the Patient Information sheet including the Declaration of Informed Consent, with the request that they reread the information carefully and if they consent to the conditions return the signed declaration when they attend the next appointment. The patients will be given the opportunity to ask the study staff further questions. Among the preconditions for participation in the study is the condition that no acute instability of the metastasized vertebral body is detected in CT recorded in the course of planning the radiotherapy regimen.

A block randomization procedure shall be used to ensure the even distribution of the patients into the two intervention groups. The patients shall then be assigned 1:1 to one of the two treatment arms by the study director (or an authorized representative) on the basis of the baseline measurements. The randomization procedure shall be carried out by a central office. The study personnel responsible for the recruitment and baseline measurements shall have no access to the randomization list, and the study director no influence on the recruitment of the patients. The recruitment phase shall be concluded with the attainment of the planned number of patients (60 patients in total). It shall last twelve months, and is scheduled to start in September 2011. Regular study participation shall end two years after enrolment into the study or, where applicable, with the respective patient's death.

### Inclusion criteria

• Patients with a histologically secured tumour diagnosis, with secondarily diagnosed solitary/multiple metastatic processes in the thoracic or lumbar spine or in the os sacrum

• Indication for radiotherapy of the osseous metastatic processes

• Age: between 18 and 80 years

• Karnofsky index [12,13] ≥ 70

• Signed Declaration of Informed Consent

• Bisphosphonate therapy

• No risk of fracture indicated by prior assessment performed at t_0 _(the assessment of the instability shall be made independently by a radiologist and a specialist for A&E surgery/orthopedic specialist; two positive votes are required)

### Exclusion criteria

• Present vertebral-body instability

• Significant neurological or psychiatric disorders, including dementia and epileptic seizures

• Other severe disorders that in the judgement of the study director may prevent the patient's participation in the study

• Lacking or diminished legal capacity

• Any medical of psychological condition that the study director considers a preventive factor for the patient's ability to complete the study or to adequately understand the scope of the study and to give his/her consent

### Intervention group

#### Sport concept: differentiated isometric exercise of the autochthonous muscles

1. Exercises in the "all-fours" position:

Starting position: Arms and upper legs perpendicular to the floor, hands and knees positioned vertically under the shoulders and hips, respectively. The spine is as far as possible in the zero position over all segments. The elbows are slightly flexed. From the starting position, the right arm is anteverted in the shoulder from the sagittal level in the ventrocranial direction, at the very most until it is horizontal. The arm is then dropped again until it reaches the starting position, but it does not touch the ground again until the series of exercises is ended. The patient should keep his/her spine completely stable while the arm is being moved. The exercise is then repeated with the left arm. Duration: two series per arm, repeating each series of exercises ten times.

2. Exercises in the "gluteus arch" position:

Starting position: Lying on the back, the feet on the floor drawn up to the trunk, the knees well bent, the arms lying relaxed next to the body, the spine in the zero position as far as physiologically possible. The patient then pushes his/her posterior and back up from the floor. This movement should be made until an extension/flexion zero position in the hip is achieved. This caudal movement should take place exclusively in the hip, with the spine remaining in its physiological zero position in the lumbar and thoraco-lumbar and in the lower thoracic sections, with flexion occurring only at the point of contact in the upper thoracic segments. After reaching the highest point of the movement, the patient then lowers the trunk again until it almost reaches the starting position, and then repeats the movement without allowing the trunk to touch the floor. Duration: two series, repeating the exercise ten times in each series. Intervals between series: 60 to 90 seconds.

3. Exercises in the supine position:

Starting position: Lying on the back, the feet on the floor drawn up to the trunk, the arms lying relaxed next to the body, the spine in the zero position as far as physiologically possible. The patient tips his/her pelvis at the sagittal level in the dorsal direction, resulting in a delordosing of the lumbar spine. This effect is produced by a dorsal displacement of the two spinae iliacae posteriores superiores and a caudal displacement of the os sacrum. The patient should maintain this position of the pelvis and the lumbar spine, and then lift his/her leg, drawing the knee in the direction of the abdomen, with the other leg following suit. The legs are then very slowly moved in the caudal direction and subsequently drawn back towards the abdomen. Duration: two series, repeating the movement four to eight times in each series. Intervals between series: 60-90 seconds [14,15].

### Control group

#### Physical measure: hot towel rolls with essential oils

The physical treatment of the patients in the control group shall take the form of the ventro-thoracic application of a so-called "hot roll", a physiotherapeutic measure. For this procedure, a suited towel is rolled up and hot water poured on it so that all layers are thoroughly moist. Essential oils can also be used, which then unfold their effect as a result of the evaporation of the hot water. The patient either sits or lies on his/her back, and the rolled towel is carefully pressed onto his/her thorax, resulting in a considerable warming effect in this area. The therapist gradually unfolds the roll one layer at a time, dabbing the patient's skin with each warm layer of the towel in the process. The patient is asked to comment on his/her comfort at regular intervals to ensure that the skin is not overheated; the patient should be as relaxed as possible at all times, inhaling deeply to benefit from the respiratory-therapeutic effect of the essential oils. Duration: approx. 15 minutes.

#### Questionnaire diagnostics

The secondary endpoints such as fatigue, quality of life, and anxiety shall be recorded using validated questionnaires (EORTC QLQ C30 FA13 [16], EORTC QLQ C30 BM22 [17] and the questionnaire to record stress in cancer patients (FBK) acc. to Herschbach [18] (t_0, 2, 3_). Furthermore, all patients will also be asked to record their pain history using a pain diary (documentation of medication daily during treatment, once weekly after the end of treatment, VAS pain scale).

#### Laboratory diagnostics

The blood samples taken at the recording intervals t_0 _(baseline examination first day of radiotherapy) and t_2 _(12 weeks after completion of radiotherapy) (30 ml from a cubital vein) shall be processed within a time of max. two hours (ELISA and functional analysis) or deep-frozen at a temperature of -70°C for analysis at a later time. The samples shall be analyzed at the Dr. Limbach Laboratory in Heidelberg. The blood and urine samples shall be taken at the t_0 _and t_2 _sampling times. The following parameters shall be analyzed: NTX (N-telopeptides from collagen I), CTX (carboxy-terminal collagen crosslinks), PINP (procollagen (I) N-terminal propeptide), DPD-crosslinks (deoxypyridinoline crosslinks), and Bone aP (bone-specific alkaline phosphatase).

#### Assessment of the therapeutic success

The aim of the study is to evaluate the feasibility of the training programme described here. Progression-free and fracture-free survival, improved response to radiotherapy by means of bone density, and clinical parameters such as pain, quality of life, and fatigue constitute secondary study objectives. In addition, the changes between baseline and Week 12 and Week 24 (end of intervention) regarding pain symptoms between the intervention arm (muscle exercises) and the control arm (physiotherapy) shall be compared. The feasibility shall be expressed in percent in tabular form and shall cover the complete performance of the training programme up to the t_2 _interval. As described above, the patients enrolled into the study shall be subjected to a CT screening of the vertebral column with bone densimetry as per the standards of the follow-up investigation (t_2_). Furthermore the psycho-oncological parameters on t_1, 2, 3 _and the laboratory parameters on t_0 _and t_2 _shall be documented and evaluated. Following the radiotherapy period the patients shall independently keep a pain diary and a record of their training exercises. No further radiological examinations shall be conducted in the course of this study.

#### Clinical examinations

The baseline examination shall be carried out on the first day of radiotherapy prior to the start of therapy and is scheduled to comprise the comprehensive recording of the sociodemographic data, the recording of the current pain situation, the fear of suffering fractures, the quality of life, the current degree of ability to go about day-to-day activities, and fatigue, and shall also involve the taking of blood and urine samples. The follow-up examinations shall take place after the end of radiotherapy (day of the last fraction) and twelve weeks and six months after radiotherapy, measuring those parameters recorded at the baseline examination. The further follow-up examinations shall correspond to those carried out as standard after-care investigations.

#### Therapy drop-out criteria

• At the patient's wish

• Medical condition requiring the discontinuation of therapy in the opinion of the study director or patient

• Insufficient compliance

#### Study discontinuation

• Medical or ethical aspects that make it impossible to continue the study

• Difficulties in recruiting participants that involve an unreasonable prolongation of the study duration

• Adverse reactions that have not yet been reported in their form, severity, duration, and impact

• Unexpectedly high incidence of already known adverse reactions

• By official decision

#### Statistical analysis

The total number of patients undergoing radiotherapy in the radiation oncology department of the Heidelberg University Clinic for metastatic processes in the vertebral column in the recruitment period is approx. 120, about 90 of whom shall fulfill the inclusion criteria. The relatively weakly distinct compliance of this group of patients notwithstanding, it should be possible to achieve the planned recruitment target within a period of six months. On account of the explorative character of this study it is not possible to estimate the total number of cases; with a scheduled number of 30 patients per group, it will, however, be possible to detect a standardized mean-value effect of 0.8 with a power of 80% and an α significance level of 5%.

#### Ethical issues, information, and safety

The study protocol, Patient Information sheet, and Declaration of Informed Consent shall be submitted to the Heidelberg University Ethics Committee responsible for the study directors for review. The study shall commence only after receipt of the approval of the Ethics Committee. The positive vote was given on the 1^st ^August 2011.

The study directors shall immediately notify the Ethics Committee of all changes made in the study protocol that may have an impact on the safety of the patients. Furthermore, the Ethics Committee shall also be notified of all severe adverse events reported to the study directors and of the regular or premature termination of the study. The procedures described in the submitted study protocol regarding the performance, evaluation, and documentation of this study have been selected in such a way that the principles of the Good Clinical Practice (GCP) guidelines are observed. The regulations regarding medical confidentiality and data protection are fulfilled.

## Discussion

Bone-affecting metastatic processes in vertebral bodies constitute a frequent secondary disorder in connection with a variety of primary tumours. Palliative percutaneous radiotherapy is one of the therapeutical options available in this connection. On the one hand, symptoms such as painful impairments of mobility, pain at rest, a fear of pathological fractures, and fatigue result in a pronounced diminution in the patients' quality of life, while on the other hand the therapy of such disorders involves protracted, cost- and time-intensive therapeutical measures. Furthermore, in some cases there is an acute risk of fracture with the danger of the emergence of symptoms of paraplegia. Our point of departure is the assumption that additional differentiated sport therapy aimed at strengthening the paravertebral muscle system can enhance the impact of radiotherapy by exerting a positive effect on clinical factors and bone density. The aim of this explorative study is to investigate the feasibility of muscle-training exercises and to evaluate the progression- and fracture-free survival time and the improvement of bone density, as well as to assess other clinical parameters such as pain, quality of life, and fatigue as secondary endpoints. A further objective of the study is to make a contribution to the integration of a regimen of physical training exercises with its multidimensional effects into future therapeutical concepts for patients with osseous metastases of the vertebral bodies.

## Competing interests

The authors declare that they have no competing interests.

## Authors' contributions

HR and JD developed the study protocol and planned the trial. TB is responsible for statistical considerations/basis of the trial. HR is responsible for conducting and co-ordination of the trial as well as patient recruitment. All authors read and approved the final manuscript.

## Pre-publication history

The pre-publication history for this paper can be accessed here:

http://www.biomedcentral.com/1471-2407/11/482/prepub
